# Prevalence of Sarcopenia and Dynapenia and Related Clinical Outcomes in Patients with Type 1 Diabetes Mellitus

**DOI:** 10.3390/nu15234914

**Published:** 2023-11-24

**Authors:** María Carmen Andreo-López, María Teresa Zarco-Martín, Victoria Contreras-Bolívar, María Luisa Fernández-Soto

**Affiliations:** 1 Endocrinology and Nutrition Unit, University Hospital Clínico San Cecilio, 18016 Granada, Spain; mcandreo21@gmail.com (M.C.A.-L.); victoriacontreras_87@hotmail.com (V.C.-B.); mlfernan@ugr.es (M.L.F.-S.); 2 Instituto de Investigación Biosanitaria de Granada (Ibs. Granada), 18012 Granada, Spain; 3 CIBER on Frailty and Healthy Aging (CIBERFES), Instituto de Salud Carlos III, 18012 Granada, Spain; 4 Department of Medicine, University of Granada, 18016 Granada, Spain

**Keywords:** type 1 diabetes mellitus, sarcopenia, dynapenia

## Abstract

Background: Sarcopenia has recently been recognized as a complication of diabetes. However, there are few results about the prevalence of sarcopenia and dynapenia and the related clinical outcomes in type 1 diabetes mellitus (T1DM). Our objectives were to evaluate the prevalence of sarcopenia and dynapenia and to determine whether there are any associations with disease-related factors in people with T1DM. Methods: A cross-sectional study was conducted in people with T1DM. We assessed appendicular skeletal mass index (ASMI) using bioimpedance 50 Hz (Nutrilab Akern). Muscle function was assessed through handgrip strength (HGS) using a Jamar dynamometer. Sarcopenia was defined as a low HGS with low ASMI, whereas dynapenia was defined as low HGS with a normal ASMI. We used HGS data from the Spanish population percentile table and a cut-off point at p5 as dynapenia. The association of clinical, metabolic, and lifestyle variables with sarcopenia and dynapenia was studied. Results: This study included 62 T1DM patients (66% females, mean age of 38 ± 14 years, body mass index (BMI) of 24.9 ± 4.7 kg/m^2^). The prevalence of sarcopenia and dynapenia was 8% and 23%, respectively. In our sample, there were more men in the sarcopenic and dynapenic groups. The sarcopenic group showed a significantly higher mean HbA1c value. Lower diabetes duration, PREDIMED score, BMI, and muscle mass measures (fat-free mass index (FFMI), ASMI, and body cell mass index (BCMI)) were significantly associated with sarcopenia. Decreased diabetes duration, PREDIMED score, phase angle (PhA), and HGS values showed a significant association with dynapenia. Conclusions: The prevalence of sarcopenia and dynapenia was high in people with T1DM in our study. Specifically, the proportion of dynapenia was quite high. HGS and ASMI are practical tools for the assessment of muscle health status in T1DM, and low values are associated with poor glycemic control, underweight, and low adherence to the Mediterranean diet. Thus, dynapenia may predict accelerated muscle aging in T1DM.

## 1. Introduction

T1DM is a chronic endocrine disease characterized by insulin deficiency due to autoimmune destruction of the β-cells of the pancreas in most cases [1]. The global prevalence of T1DM is 6 per 10,000 people, and the incidence of T1DM has risen rapidly over the last 50 years. A recent paper estimated a prevalence of 15 per 100,000 people per year [2].

Poor glycemic control may promote a variety of complications in people with diabetes [1]. In fact, T1DM has often been associated with macrovascular and microvascular complications, such as retinopathy, neuropathy, and diabetic kidney disease [3]. However, skeletal muscle dysfunction may be a complication of T1DM. Some authors consider diabetic sarcopenia to be a primary complication of diabetes rather than neuropathy [4,5]. In fact, Monaco et al. suggested T1DM as an accelerator of muscle aging [6]. Hence, sarcopenia in people with diabetes has received special attention in recent years. Moreover, sarcopenia can impact the physical and metabolic well-being of patients with T1DM, increasing the risk of mortality [7]. Dynapenia may even contribute to the development of osteoporosis, reported in adults with diabetes [8]. Hence, it could be interesting to detect and diagnose dynapenia and sarcopenia.

The most recent studies report the prevalence of sarcopenia among type 2 diabetes mellitus (T2DM). These studies were carried out on different populations (such as different sexes and age groups) and the prevalence of sarcopenia ranged between 7.2% and 31.1% [5,9,10,11,12]. The prevalence of dynapenia in European T2DM was even remarkable in people with diabetes: 39.3% vs. 18% of people without diabetes [13]. Even though poor glycemic control and insulin therapy are reported to be associated with sarcopenia in T2DM, T1DM may be a stronger risk factor for sarcopenia [14]. In fact, in a recent study in Japanese people over 65 years of age, a prevalence of sarcopenia and dynapenia of 42% and 12% was observed in the T1DM population, compared to 21% and 14% in patients with T2DM. Specifically, the prevalence of sarcopenia and dynapenia in an older European sample of 33 people with T1DM was reported to be 10.3% and 7.7%, respectively [15]. However, the prevalence of sarcopenia and dynapenia has not been studied in European individuals <40 years of age. Moreover, young people are less likely to suffer from age-related muscle dysfunction. Thus, the chances of linking sarcopenia to T1DM are increased.

Our objective was to evaluate the prevalence of sarcopenia and dynapenia in T1DM. Moreover, we aimed to determine if there are any associations with clinical outcomes in people with T1DM.

## 2. Materials and Methods

### 2.1. Study Design

A cross-sectional observational study with sequential recruitment of people with T1DM who attended the outpatient consulting room for their follow-up examination was conducted.

All participants met the following inclusion criteria—patients diagnosed with T1DM who agreed to participate in the study via accepted informed consent—and none of the following exclusion criteria: patients of pediatric age, pregnant, or breastfeeding. All subjects provided informed consent before participating in the study, which was reviewed and approved by the Regional Ethics Committee.

### 2.2. Clinical Variables

We collected data on sex, age, family history of diabetes, diabetes duration, diagnosis of autoimmune diseases, hospital admissions for ketoacidosis, and micro- and macrovascular complications.

### 2.3. Lifestyle Parameters

Dietary habits were assessed with the 14-item PREDIMED questionnaire [16]. This questionnaire assesses adherence to the Mediterranean diet (MedDiet), considering a cut-off point to good adherence ≥9. Physical activity was assessed with the International Physical Activity Questionnaire (IPAQ) [17], which evaluates the activity performed in the last 7 days, classifying it into vigorous, moderate, walking, and sitting time.

### 2.4. Anthropometric Measurements

Weight (kg) was assessed using a scale (SECA, Birmingham, UK), and height (m) was obtained using a stadiometer (SECA, Birmingham, UK). With these two values, body mass index (BMI) was calculated (kg/m^2^).

### 2.5. Body Composition Parameters

Body composition analysis was obtained using Nutrilab, a 50 kHz phase-sensitive impedance analyzer (Bioimpedance Vector Analyzer (Akern, Florence, Italy [18]). The phase angle (PhA) was expressed in degrees as arctan (Xc/R) × (180°/*p*). An individual standardized PhA value (SPhA) was determined by adjusting by sex and age. Data obtained using BIA for body composition were categorized as BMI, fat-free mass (% FFM), fat mass (% FM), total body water (% TBW), extracellular water (% ECW), body cellular mass index (BCMI, kg/m^2^), skeletal muscle mass index (SMI, kg/m^2^), and appendicular skeletal muscle mass index (ASMI, kg/m^2^), obtained from predictive equations [19,20]. The fat-free mass index (FFMI, kg/m^2^) was also calculated: FFM (kg)/height (m^2^).

### 2.6. Muscle Strength

Muscle strength was assessed using an adult dynamometer (Jamar handgrip dynamometry, Asimow Engineering Co., Los Angeles, CA, USA) and was performed in the non-dominant limb, repeated on three occasions, and the mean was recorded. The mean was calculated and, along with the highest value, was used to represent HGS.

To classify normality, data from the Spanish population were used, establishing a cut-off point at p5. Data are expressed in absolute figures and compared to the reference population [21].

### 2.7. Biochemical and Metabolic Variables

The biochemical variables included fasting blood glucose (FBG) (mg/dL), total cholesterol (mg/dL), LDL cholesterol (mg/dL), HDL cholesterol (mg/dL), triglycerides (mg/dL), albumin (g/dL), prealbumin (mg/dL), and C reactive protein (CRP) (mg/dL).

The metabolic variables included glycated hemoglobin A1c (HbA1c) (%) obtained by the biochemical analysis, daily total dose insulin (TDI, IU), daily total dose insulin per kilogram (TDI/kg), and insulin sensitivity factor (ISF) calculated by 1650/TDI.

### 2.8. Sarcopenia Diagnosis

Sarcopenia was defined as low muscle strength (dynapenia) and low muscle mass (myopenia). Dynapenia criteria were defined in Section 2.6. Myopenia was assessed by ASMI using BIA. The cut-off points used were the ones proposed by the European Working Group on Sarcopenia in Older People 2 (EGWSOP2) (men < 7 kg/m^2^, women < 5.5 kg/m^2^) [22].

### 2.9. Statistical Analysis

Data analyses were carried out using IBM SPSS 25.0 (IBM, New York, NY, USA), and graphic representation was performed using R v.3.5.1 software (Integrated Development for R. RStudio, PBC, Boston, MA, USA).

Normality of the distribution of quantitative variables was verified using the Shapiro–Wilk test. Quantitative variables are presented as the mean and standard deviation, and differences between paired observations were determined using the Student’s *t*-test (or the Wilcoxon test in the absence of normality). The qualitative variables are expressed as proportions, and the differences between groups were analyzed via the Chi-square test, using Fisher’s exact test when necessary.

Pearson correlation coefficients between the variables were obtained. Logistic regression analysis was used to calculate the cross-sectional association of dynapenia and sarcopenia using as independent variables those with significant differences in the Student’s *t*-test and clinical relevance. The model was then adjusted for sex and age. Statistical significance was set at *p* < 0.05.

## 3. Results

**Figure 1:**
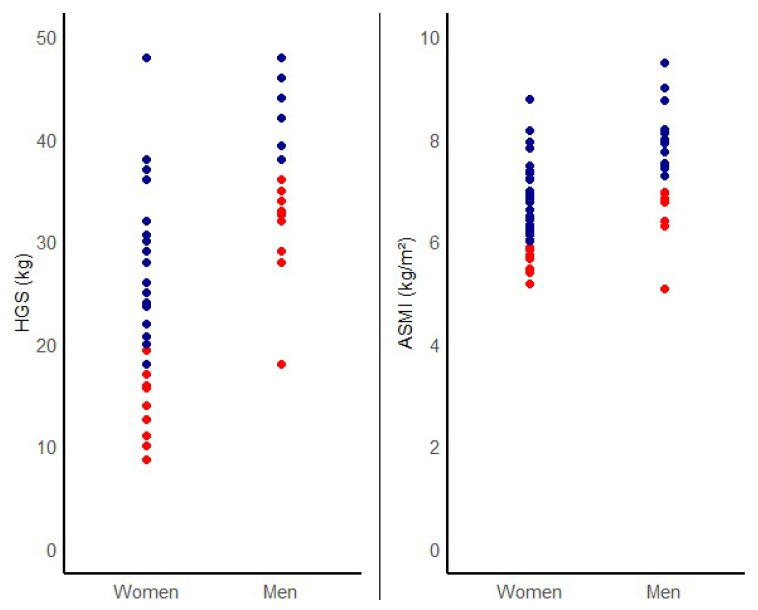
Individual data from HGS and ASMI divided by sex (blue points indicate normal values; red points indicate low values according to the reference population).

A total of 62 patients with T1DM were recruited: 21 (34%) males and 41 (66%) females. The mean age was 38 ± 14 years, and the general characteristics are displayed in Table 1. The mean duration of diabetes was 21 ± 14 years. The prevalence of microvascular complications was 26%, and none of the patients had macrovascular complications. The prevalence of a family history of diabetes was 58%, 38% had hospital admissions for ketoacidosis during the course of the disease, and 34% had other autoimmune diseases. The mean of A1c was 8.4 ± 1.5%, and only 27% of patients reached the glycemic target (HbA1c ≤ 7%). The prevalence of dynapenia was 23% and that of sarcopenia was 8%. Of the sample, 6.5% (4) had low ASMI with normal HGS. Values from ASMI and HGS divided by sex are shown in Figure 1.

### 3.1. Clinical Characteristics of Sarcopenia and Dynapenia

Males showed a higher prevalence of sarcopenia than females. Sarcopenic patients with T1DM had a significantly lower diabetes duration, PREDIMED score, and BMI than those without sarcopenia. HbA1c was also significantly higher in the sarcopenic group (Table 1).

In relation to body composition, we found significantly lower levels in the sarcopenic group compared to the non-sarcopenic group with respect to FM, FFMI, BCMI, and ASMI and significantly higher levels in TBW (Table 2).

No differences were found between the groups in terms of age, microvascular complications, IPAQ, metabolic and biochemical variables, phase angle, or HGS.

Concerning the dynapenic group, they showed a higher prevalence in the group of males. The T1DM patients with dynapenia had significantly lower means in diabetes duration, PREDIMED score, PhA, and SPhA and higher values for ECW than the non-dynapenic group (Table 1 and Table 2).

### 3.2. Correlation between Study Variables

**Figure 2:**
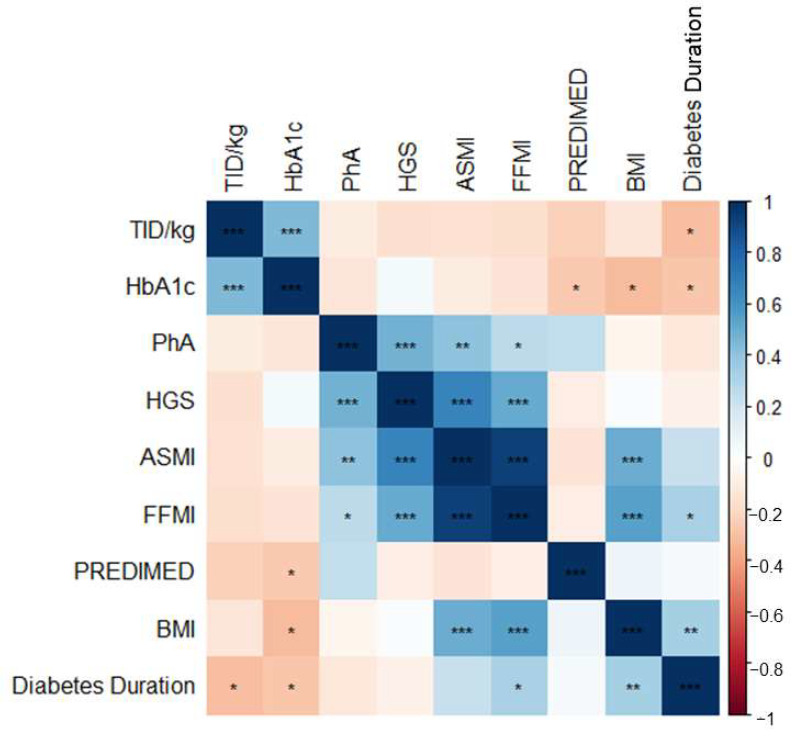
Correlation matrix plot. * *p*-value < 0.05, ** *p*-value < 0.01, *** *p*-value < 0.001.

Regarding glycemic control, HbA1c had a positive significant correlation with TID/kg (r = 0.46, *p* < 0.001) and a negative significant correlation with time disease evolution (r = −0.28, *p* = 0.03), BMI (r = −0.32, *p* = 0.01) and PREDIMED score (r = −0.26, *p* = 0.04) (Figure 2).

Concerning diabetes duration, it had a negative correlation with TID/kg (r = −0.31, *p* ≤ 0.02). Moreover, it was positively correlated with FFMI (r = 0.33, *p* = 0.01) and BMI (r = 0.34, *p* = 0.01).

HGS was positively correlated with PhA (r = 0.48, *p* < 0.001), ASMI (r = 0.66, *p* < 0.001), and FFMI (r = 0.50, *p* < 0.001).

### 3.3. Risk Factors for Sarcopenia and Dynapenia in T1DM

**Figure 3:**
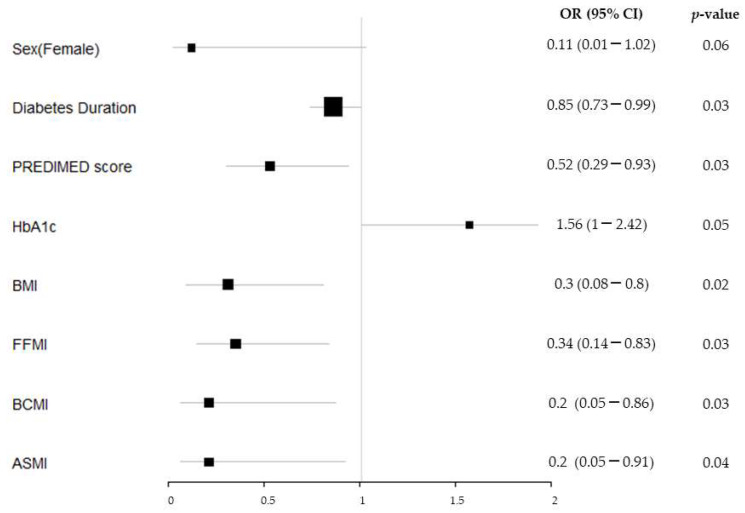
Sarcopenia odds ratio plot. The size of the black squares indicates the weight that the result of each variable has on the final result (sarcopenia or dynapenia). The bigger size, the lower variability.

Sarcopenia was significantly associated with a lower diabetes duration, PREDIMED score, BMI, FFMI, BCMI, and ASMI (Figure 3).

**Figure 4:**
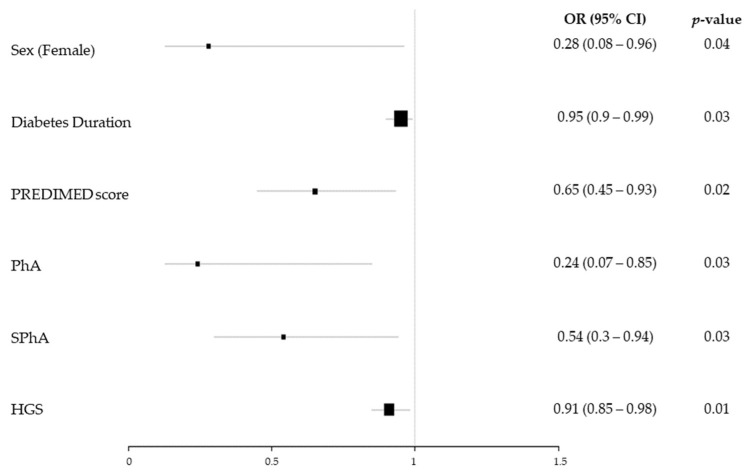
Dynapenia odds ratio plot. The size of the black squares indicates the weight that the result of each variable has on the final result (sarcopenia or dynapenia). The bigger size, the lower variability.

Dynapenia had a significant association with male sex and a lower diabetes duration, PREDIMED score, PhA, SPhA, and HGS (Figure 4).

The analysis adjusted by sex and age showed that sarcopenia was significantly associated with lower diabetes duration (OR: 0.8 (95% CI 0.65–0.97)). Dynapenia showed a significant association with a lower diabetes duration (OR: 0.91 (95% CI 0.85–0.98)), PREDIMED score (OR: 0.68 (95% CI 0.46–0.99)), and PhA (OR: 0.12 (95% CI 0.02–0.60)).

## 4. Discussion

To the best of our knowledge, this is the first study to assess sarcopenia and dynapenia in a young European population. We found that the prevalence of sarcopenia and dynapenia is high. Specifically, the percentage of dynapenia is three times higher than sarcopenia in this young T1DM population. In turn, there are clinical outcomes related to the disease and lifestyle of patients that may accelerate the development of diabetes-related sarcopenia in people with T1DM.

Traditionally, sarcopenia was considered an aging-related disease [10,12]. In this sense, most sarcopenia prevalence studies are usually carried out on older people. In fact, the prevalence of sarcopenia was 1% in Europeans over 60 years of age using the EWGSOP 2 [23]. However, sarcopenia seemed to go beyond age because our sample population was considerably young. In particular, some authors have pointed to T1DM as a contributing factor to muscle aging in young people [6]. In fact, T1DM is associated with alterations of the musculoskeletal system already in adolescence [8]. 

On the contrary, some studies have reported that autoimmune diseases are closely associated with sarcopenia [24]. Our sample had T1DM, which is an autoimmune disease, so it is possible that this pathophysiological phenomenon is associated with muscle dysfunction.

In our study, the prevalence of sarcopenia was 8%, lower than that reported in other studies [14,25]. However, the average age was younger in our sample. Mori et al. detected a prevalence of sarcopenia of 16.6% in 36 people aged >55 years with T1DM [25]. In the same line, in another study conducted in older people (≥65 years), the prevalence was 20% [14].

Sarcopenia can impact the physical and metabolic well-being of patients with T1DM. It may even increase the risk of mortality [7]. Specifically, we found that our prevalence of dynapenia was 23% higher than the prevalence of low ASMI or myopenia. Thus, the deterioration of muscle strength may precede the reduction in muscle mass in our patients with T1DM, consolidating the results of previous studies [26]. Similarly, a decrease in muscle strength of almost twice as much was observed in the population with diabetes compared to the population without diabetes [27].

On the contrary, some of our clinical outcomes are related to the development of sarcopenia in T1DM. Some of them were patient-dependent, such as sex, adherence to a MedDiet, and BMI. In contrast, other clinical results were T1DM-dependent, such as disease time evolution or HbA1c.

Previous studies found that sarcopenia was more prevalent in males than in females with diabetes [5]. In our study, male sex was associated with sarcopenia. Similarly, in Feng’s meta-analysis, a slightly higher prevalence of sarcopenia was detected in men. These findings were linked to a possible decrease in testosterone secretion [5]. This could explain the faster decline in the rate of male skeletal muscle than in women [5]. Nevertheless, Okamura et al. found that men have a reduced risk of sarcopenia [28]. Therefore, both men and women with diabetes should be screened for sarcopenia.

With respect to diet, it is considered a patient-modifiable factor. Specifically, the MedDiet is considered a good strategy in the management of young people with T1DM [29]. In our study, people with sarcopenia showed a lower PREDIMED score. Similarly, evidence is accumulating on the beneficial association between MedDiet and muscle mass/function [30,31,32]. In addition, we observed that dynapenia was related to a lower PREDIMED score in this study. In this regard, several works have associated the MedDiet with HGS [30,33]. The MedDiet is rich in fruits, vegetables, nuts, extra virgin olive oil, and fish. These foods have antioxidant properties that are positively related with HGS or skeletal muscle strength [34,35,36,37,38].

In terms of muscle mass, our sarcopenic group had worse PREDIMED scores and lower FFMI and ASMI values. Some studies have established a positive association between MedDiet and muscle mass [37,39]. Specifically, Cervo et al. positively associated the MedDiet with appendicular lean mass [39].

Moreover, an antidiabetic effect has been described due to bioactive constituents (e.g., polyphenols) in multiple MedDiet foods. Therefore, this diet could improve metabolic control. In our study, the MedDiet was negatively correlated with HbA1c. In addition, our sarcopenic group had lower PREDIMED scores and poorer metabolic control or HbA1c compared to the non-sarcopenic group.

With regard to metabolic control, we found a negative correlation between HbA1c, FFM, and BMI. In fact, some works have associated chronic hyperglycemia with the deterioration of skeletal muscle in T1DM [25]. In this relationship, the accumulation of advanced glycosylation products appears key [25]. We also found that higher HbA1c levels were associated with higher insulin requirements. In turn, we observed in the sarcopenic group lower BMI, FFM, and ASMI and poorer HbA1c. In fact, it has been reported that insulin deficiency increases the risk of sarcopenia [4]. Indeed, insulin and insulin-like growth factor (IGF-1) are anabolic factors for skeletal muscle. Insulin stimulates the growth factors involved in myogenesis [40] and is essential for the proper turnover of skeletal muscle proteins [41,42]. Conversely, insulin deficiency may promote underweight and malnourishment in our sarcopenic group. Nevertheless we cannot claim that people with T1DM in the sarcopenic group were malnourished because we did not carry out the diagnosis according to the latest consensus proposed by the GLIM (Global Leadership Initiative on Malnutrition) [43].

Moreover, assessing the nutritional status of patients with T1DM, it may be useful to perform body composition analysis beyond BMI. We used BIA because it allows us to obtain the phase angle, a biomarker for malnutrition and a predictor for morbimortality [44]. Specifically, our study showed in the dynapenic group lower PhA and SPhA values. In line with these results, Yamada et al. revealed that the sarcopenic and dynapenic groups had lower PhA values in the older population [45]. However, no comparable T1DM evidence exists. This enhances the value of PhA not only as a marker of muscle mass, but also of strength and function. Moreover, some studies have shown that populations with T1DM have a lower phase angle compared to healthy control groups. This may be due to the fact that hyperglycemia can induce a moderate osmotic effect by disrupting the intra–extracellular water ratio [46,47]. According to this, our sample showed higher TBW in the sarcopenic group and higher ECW in the dynapenic group. This could be a reflection of the poor glycemic control of the sample, especially in the sarcopenic group.

On the contrary, our results showed a shorter diabetes duration in the sarcopenic and dynapenic groups. Conversely, previous studies recognized the duration of diabetes as a risk factor for sarcopenia [5,48,49,50,51]. Nonetheless, these studies were conducted in patients older than 60 years with T2DM. However, our sarcopenic population was younger and thinner in contrast to the typical T2DM phenotype. In addition, it was highlighted that years of disease progression were correlated negatively with HbA1c and positively with BMI and total insulin dose per kilogram. Once more, our findings suggest that sarcopenic patients and shorter T1DM evolution time may be insulin-deficient. Perhaps our non-sarcopenic group had more experience in diabetes management and advanced knowledge because their mean age of disease was 20 years.

Regarding dynapenia, we found few studies that related T1DM evolution time with muscle strength. Nevertheless, previous studies observed that worse metabolic control contributed to reduced strength, and we found a correlation between HbA1c and disease evolution years. In fact, accumulated advanced glycation end-product (AGE) was associated with dynapenia in patients with T2DM [11]. Moreover, the accumulation of AGEs in fast twitch muscle fibers cross-links muscle collagen and reduces the tonic force of muscle contraction [52,53]. This mechanism could be involved in the impaired muscle function in people with T1DM [54].

Moreover, people with T1DM and poorly maintained metabolic control often present chronic complications. Namely, those with diabetic polyneuropathy (DPN) were characterized by both higher fatigability and lower muscle strength [55]. However, recent studies have provided strong evidence that those with T1DM exhibit muscle-specific alterations, even in the absence of neurologic complications [27,56]. In fact, this study did not establish an association between sarcopenia and DPN.

Concerning diabetic nephropathy, we did not find significant differences between groups with and without sarcopenia. However, previous studies in T2DM found an association between sarcopenia and albuminuria in those under 60 years of age [57].

Similar to our results, the accumulating evidence does not demonstrate a direct relationship between diabetic retinopathy and sarcopenia/strength [58]. However, in a study in T2DM, proliferative diabetic retinopathy in progression was linked to the risk of sarcopenia and HGS [59]. Specifically, in our work, no differentiation was made between proliferative and non-proliferative retinopathy.

This study has some strengths. This was the first study that focuses on sarcopenia in a European population aged <40 years with T1DM. In addition, the reference tables for low handgrip strength were used according to our population (adapted to demography, sex, and age). Our sample was young and the findings could be of interest for the prevention of sarcopenia.

Nonetheless, our study has limitations, as the study design did not allow us to establish causality. The sample was not homogeneous and the results should be interpreted with caution. Moreover, testosterone levels have not been studied in males and may lead to sarcopenia in this subgroup. Some microvascular complications of diabetes could also have been misdiagnosed.

Given the growing interest and the harmful consequences of sarcopenia, more studies are needed to strengthen the possible link between sarcopenia and diabetes in order to understand the risk factors in people with T1DM and to be able to prevent them.

## 5. Conclusions

The prevalence of sarcopenia and dynapenia was high in patients with T1DM in our study. The proportion of dynapenia was higher than the prevalence of low ASMI or myopenia. Moreover, HGS and ASMI are practical tools for the assessment of muscle status in T1DM, and low values reflect poor metabolic status and are associated with poor glycemic control, low adherence to the MedDiet, and underweight.

Moreover, dynapenia may manifest as a form of accelerated muscle aging, or “a pre-sarcopenic stage”. Muscle strength was also adversely affected in patients with T1DM developed independently of other diabetic complications.

## Figures and Tables

**Table 1:** Baseline characteristics of the population and according to sarcopenia and dynapenia diagnosis.

	Total (*n* = 62)	No Sarcopenia (*n* = 57)	Sarcopenia (*n* = 5)	*p*-Value	No Dynapenia (*n* = 48)	Dynapenia (*n* = 14)	*p*- Value
Clinical variables							
Sex				0.02			0.04
Male	21 (33.9)	17 (29.8)	4 (80)		13 (27.1)	8 (57.1)	
Female	41 (66.1)	40 (70.2)	1 (20)		35 (72.9)	6 (42.9)	
Age (years)	38 ± 14	39 ± 14	36 ± 19	0.83	37.4 ± 13.6	38.5 ± 16.9	0.81
Diabetes duration (years)	20.5 ± 13.9	21.9 ± 13.5	14.8 ± 8.6	0.01	22.7 ± 13.9	13.2 ± 11.6	0.02
Microvascular complications							
Diabetic nephropathy	7 (11.3)	7 (12.3)	0 (0)	0.41	6 (12.5)	1 (7.1)	0.58
Diabetic polyneuropathy	13 (21)	12 (21.1)	1 (20)	0.96	10 (20.8)	3 (21.4)	0.96
Diabetic retinopathy	12 (19.4)	11 (19.3)	1 (20)	0.97	10 (20.8)	2 (14.3)	0.56
Lifestyle variables							
PREDIMED score	9 ± 2	9 ± 2	7 ± 2	0.01	9 ± 2	7 ± 2	0.01
Adherence to MedDiet	36 (58.1)	35 (61.4)	1 (20.0)	0.07	31 (64.6)	5 (35.7)	0.05
IPAQ				0.41			0.81
Low	17 (29.3)	15 (27.8)	2 (50.0)		13 (28.3)	4 (33.3)	
Moderate	33 (56.9)	32 (59.3)	1 (25.0)		26 (56.5)	7 (58.3)	
High	8 (13.8)	7 (13.0)	1 (25.0)		7 (15.2)	1 (8.3)	
Metabolic and biochemical variables							
Fasting glucose (mg/dL)	156 ± 72	156 ± 70	154 ± 99	0.94	158 ± 72	147 ± 72	0.60
HbA1c (%)	8.4 ± 1.5	8.2 ± 1.7	10.0 ± 2.4	0.03	8.2 ± 1.7	8.7 ± 2.2	0.37
TID (UI)	46 ± 21	47 ± 22	41 ± 12	0.36	46 ± 22	48 ± 16	0.77
ISF	44 ± 21	43 ± 20	38 ± 13	0.38	44 ± 21	40 ± 20	0.48
TID/kg (UI/kg)	0.66 ± 0.30	0.66 ± 0.28	0.72 ± 0.34	0.65	0.64 ± 0.30	0.76 ± 0.29	0.14
Pre-albumin (mg/dL)	18.8 ± 3.6	19.2 ± 3.4	12.6 ± 3.6	0.08	19.2 ± 3.5	15.9 ± 4.7	0.24
CRP (mg/L)	4.7 ± 6.1	4.6 ± 6.1	5.1 ± 6.7	0.88	4.6 ± 6.1	4.9 ± 6.4	0.87
Muscle strength							
HGS (kg)	29 ± 11	30 ± 12	26 ± 8	0.41	31 ± 11	22 ± 10	0.01

Results are expressed as the mean ± SD (numeric variables) or *n* (%) (categorical variables). Abbreviations: CRP, C reactive protein; HGS, hand grip strength; ISF, insulin sensitivity factor; IPAQ, International Physical Activity Questionnaire; TID, total insulin dose; HbA1c, glycated hemoglobin A1c.

**Table 2:** Body composition characteristics according to sarcopenia and dynapenia diagnosis.

	Total (*n* = 62)	No Sarcopenia (*n* = 57)	Sarcopenia (*n* = 5)	*p*-Value	No Dynapenia (*n* = 48)	Dynapenia (*n* = 14)	*p*-Value
BIA and body composition variables							
Rz (Ohm)	568.6 ± 78.4	561.2 ± 75.6	650.2 ± 66.7	0.01	565.8 ± 74.5	577.9 ± 92.6	0.61
Xc (Ohm)	57.7 ± 8.1	57.7 ± 8.1	64.4 ± 4.2	0.05	58.5 ± 7.4	55.4 ± 9.9	0.21
PhA (°)	5.8 ± 0.7	5.8 ± 0.7	5.7 ± 0.3	0.56	5.9 ± 0.6	5.5 ± 0.7	0.02
SPhA	0.4 ± 1.4	0.5 ± 1.5	−0.3 ± 0.5	0.23	0.6 ± 1.5	−0.3 ± 0.8	0.01
TBW (%)	53.6 ± 7.9	53.1 ± 8.0	59.6 ± 1.3	<0.001	52.8 ± 8.1	56.2 ± 6.6	0.16
ECW (%)	46.7 ± 3.0	46.6 ± 3.1	47.3 ± 1.8	0.66	46.2 ± 2.8	48.4 ± 3.3	0.02
FFM (%)	73.1 ± 10.4	72.3 ± 10.5	81.8 ± 1.8	<0.001	72.0 ± 10.6	76.7 ± 9.2	0.14
FFMI (kg/m^2^)	17.7 ± 2.0	17.9 ± 1.9	15.4 ± 1.3	0.01	17.8 ± 1.8	17.5 ± 2.6	0.71
FM (%)	26.9 ± 10.4	27.7 ± 10.5	18.2 ± 1.8	<0.001	28.0 ± 10.7	23.3 ± 9.2	0.14
BCMI (kg/m^2^)	9.3 ± 1.3	9.5 ± 1.3	8.0 ± 0.9	0.02	9.5 ± 1.4 1.2	5.9 ± 1.5	0.17
BMI (kg/m^2^)	24.9 ± 4.7	25.3 ± 4.5	18.8 ± 1.8	0.003	25.3 ± 4.5	23.3 ± 5.1	0.16
SMI (kg/m^2^)	8.6 ± 1.5	8.6 ± 1.0	7.8 ± 0.8	0.26	8.5 ± 1.4	8.6 ± 1.6	0.91
ASMI (kg/m^2^)	6.9 ± 1.0	7.0 ± 1.3	5.9 ± 0.9	0.03	6.9 ± 0.8	6.7 ± 1.2	0.49

Results are expressed as the mean ± SD. Abbreviations: ASMI, appendicular skeletal mass index; BMI, body mass index; BCMI, body cell mass index; BIA, bioelectrical impedance analysis; ECW, extracellular water; FFM, fat-free mass; FM, fat mass; PhA, phase angle; SMI, skeletal muscle index; SPhA, standardized phase angle; TBW, total body water; FFMI: fat-free mass index.

## Data Availability

The data presented in this study will be made available upon request to the corresponding author.
